# A case of successful endoscopic submucosal dissection of early gastric cancer involving pylorus by switching the direction of traction

**DOI:** 10.1055/a-2590-7900

**Published:** 2025-05-22

**Authors:** Takaaki Yoshikawa, Takeshi Mori, Sonoka Katsuyama, Sota Nakagami, Kenshiro Hirohashi, Shujiro Yazumi

**Affiliations:** 1 Gastroenterology and Hepatology, Medical Research Institute Kitano Hospital, PIIF Tazuke-Kofukai, Osaka, Japan

In the field of endoscopic retrograde cholangiopancreatography, internal traction is occasionally used to expose and approach the papilla 1 2 . On the other hand, pre-incision traction method is used for forming mucosal flap during endoscopic submucosal dissection (ESD) 3 . We herein present the combing traction method by switching mucosal traction to pre-incision traction method during gastric ESD involving the pylorus, which is regarded as technically difficult 4 .

We performed gastric ESD in an 81-year-old man. A 10 mm, 0-IIc lesion was on the lesser curvature of the prepylorus ( Fig. 1 ). The anal side of the lesion could not be seen. After marking the oral side, we deployed the traction to the oral, lesser curvature side using 7-rings traction (Adachi) and SureClip Eco (MC medical) ( Video 1 ). Thanks to the traction, we could see the anal side of the lesion; therefore, accomplished the rest of marking ( Fig. 2 ). Next, we used GIF-H290 (Olympus) and DualKnife J (Olympus). We turned around the endoscope in the bulb of the duodenum and accomplished mucosal incision and submucosal dissection of the anal side. We changed GIF-H290T (Olympus) and ITknife 2 (Olympus). After finishing the incision and submucosal dissection just on the pylorus, we extended the semitotal mucosal incision other than the mucosa around the traction clip. Subsequently, we only retrieved the traction clip of the oral side, and newly applied a clip to the greater curvature side as the pre-incision traction method ( Fig. 3 ). Finally, the rest of the mucosal incision and submucosal dissection was accomplished with multi-bending scope (GIF-Y0009; Olympus) and Dualknife J ( Fig. 4 ). The patient was discharged without any complications 3 days after ESD. Histopathological examination revealed that ESD was curative resection with negative margins (eCura A) ( Fig. 5 ).

Endoscopy_UCTN_Code_TTT_1AO_2AG_3AD

**Fig. 1:**
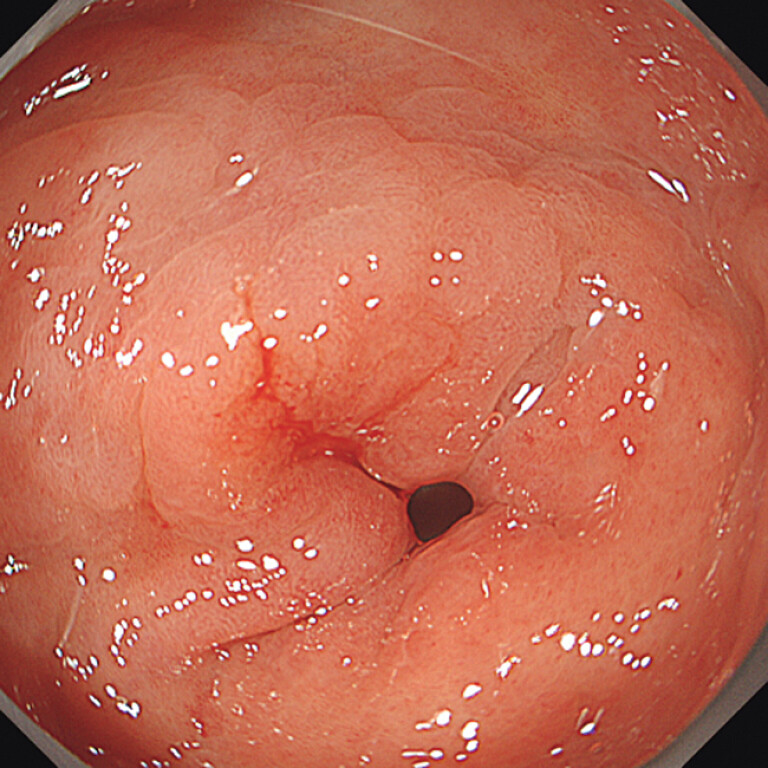
A 10 mm, 0-IIc lesion was on the lesser curvature of the prepylorus. The lesion of the anal side could not be seen.

**Fig. 2:**
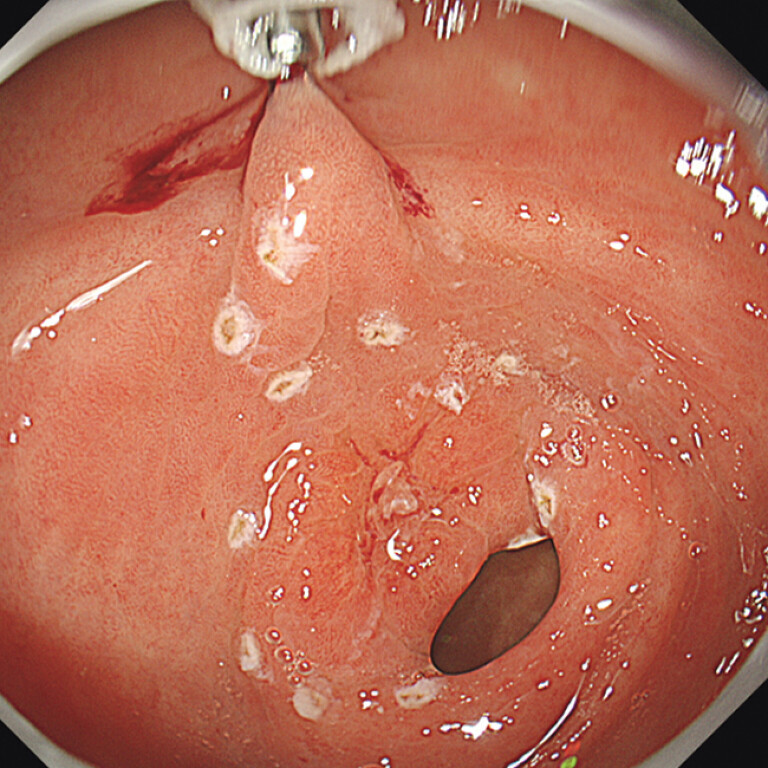
Traction to the oral, lesser curvature side made the anal side of the lesion visible.

**Fig. 3:**
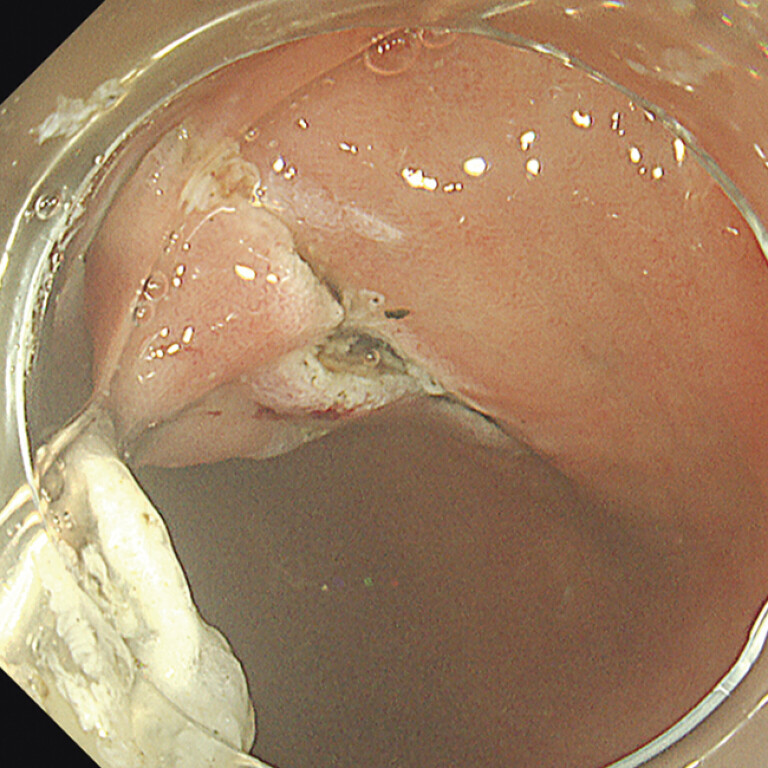
Traction to the oral, greater curvature side was used as pre-incision traction method.

**Fig. 4:**
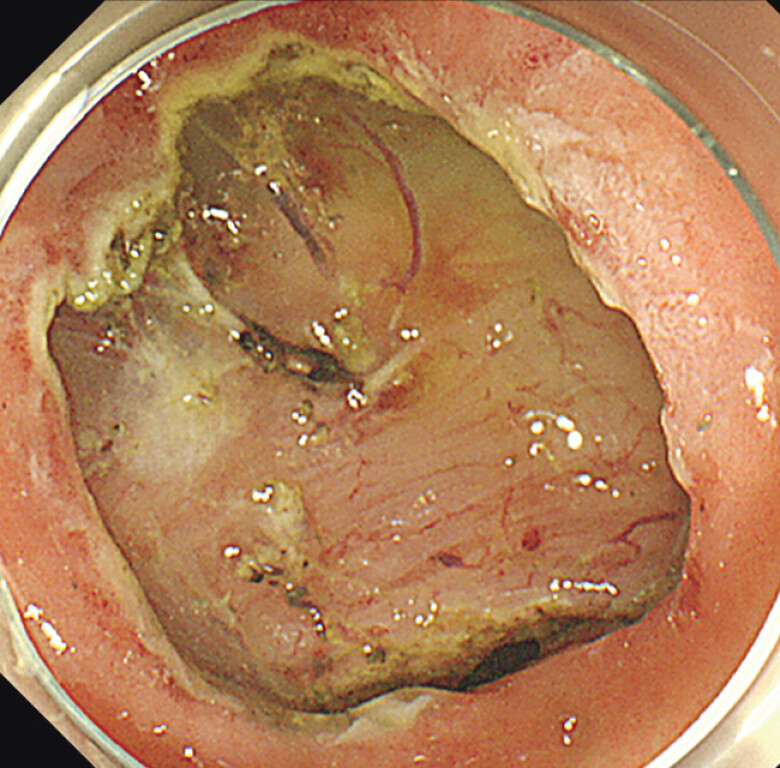
Complete resection was accomplished.

**Fig. 5:**
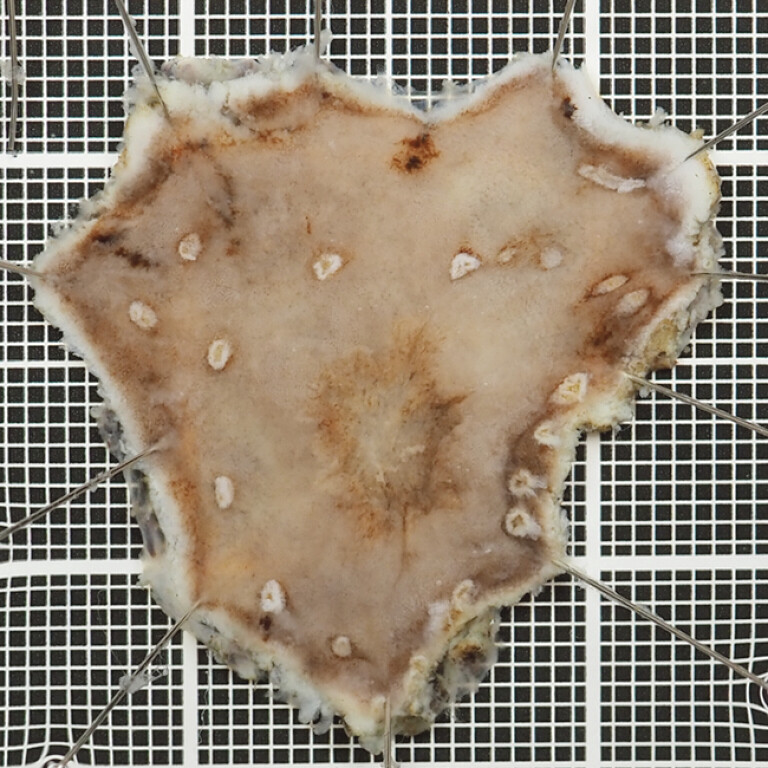
Complete resection was accomplished.

Combing traction method by switching mucosal traction to pre-incision traction method for endoscopic submucosal dissection of early gastric cancer involving the pylorus.Video 1

## Notes

Endoscopy E-Videos https://eref.thieme.de/e-videos
*E-Videos* is an open access online section of the journal *Endoscopy* , reporting on interesting cases and new techniques in gastroenterological endoscopy. All papers include a high-quality video and are published with a Creative Commons CC-BY license. Endoscopy E-Videos qualify for HINARI discounts and waivers and eligibility is automatically checked during the submission process. We grant 100% waivers to articles whose corresponding authors are based in Group A countries and 50% waivers to those who are based in Group B countries as classified by Research4Life (see: https://www.research4life.org/access/eligibility/ ). This section has its own submission website at https://mc.manuscriptcentral.com/e-videos .
